# The role of bariatric surgery to treat diabetes: current challenges and perspectives

**DOI:** 10.1186/s12902-017-0202-6

**Published:** 2017-08-10

**Authors:** Chrysi Koliaki, Stavros Liatis, Carel W. le Roux, Alexander Kokkinos

**Affiliations:** 1 First Department of Propaedeutic Internal Medicine, Diabetes Centre, Laiko General Hospital, Medical School, National and Kapodistrian University of Athens, Athens, Greece; 2 Diabetes Complications Research Centre, Conway Institute, University College Dublin, Dublin, Ireland; 3 Investigative Science, Imperial College London, London, UK

**Keywords:** Metabolic surgery, Bariatric surgery, Obesity, Type 2 diabetes mellitus, Diabetes remission

## Abstract

Bariatric surgery is emerging as a powerful weapon against severe obesity and type 2 diabetes mellitus (T2DM). Given its role in metabolic regulation, the gastrointestinal tract constitutes a meaningful target to treat T2DM, especially in light of accumulating evidence that surgery with gastrointestinal manipulations may result in T2DM remission (metabolic surgery). The major mechanisms mediating the weight loss-independent effects of bariatric surgery comprise effects on tissue-specific insulin sensitivity, β-cell function and incretin responses, changes in bile acid composition and flow, modifications of gut microbiota, intestinal glucose metabolism and increased brown adipose tissue metabolic activity. Shorter T2DM duration, better preoperative glycemic control and profound weight loss, have been associated with higher rates of T2DM remission and lower risk of relapse. In the short and medium term, a significant amount of weight is lost, T2DM may completely regress, and cardiometabolic risk factors are dramatically improved. In the long term, metabolic surgery may achieve durable weight loss, prevent T2DM and cancer, improve overall glycemic control while leading to significant rates of T2DM remission, and reduce total and cause-specific mortality. The gradient of efficacy for weight loss and T2DM remission comparing the four established surgical procedures is biliopancreatic diversion >Roux-en-Y gastric bypass >sleeve gastrectomy >laparoscopic adjustable gastric banding. According to recently released guidelines, bariatric surgery should be recommended in diabetic patients with class III obesity, regardless of their level of glycemic control, and patients with class II obesity with inadequately controlled T2DM despite lifestyle and optimal medical therapy. Surgery should also be considered in patients with class I obesity and inadequately controlled hyperglycemia despite optimal medical treatment.

## Background

Type 2 diabetes mellitus (T2DM) is associated with obesity and multiple metabolic derangements, leading to increased morbidity, mortality and financial burden. Although population-based efforts through lifestyle interventions are essential to prevent and deal with the parallel epidemics of obesity and T2DM, only few patients who have already developed T2DM and obesity are able to comply and accomplish long term weight loss and glycemic control [1].

Given its role in metabolic regulation, the gastrointestinal tract constitutes a biologically and clinically meaningful target to treat T2DM, especially in light of accumulating experimental and clinical evidence that surgery with gastrointestinal manipulations might result in T2DM remission [2]. Surgical operations with intestinal diversion and mainly duodenal-jejunal exclusion, have consistently shown beneficial effects on glucose homeostasis by reducing insulin resistance and increasing insulin secretion [3]. Mechanistic evidence further suggests that the bypass or exclusion of the duodenum and jejunum (proximal gut) may exert direct beneficial effects on glycemic control beyond those mediated by weight loss [4]. The widely used term “metabolic surgery” applies to those types of weight loss surgery modalities involving an anatomical bypass of the upper gastrointestinal tract and a functional remodelling of the intestine, which has been shown to confer the most important benefits with regard to glucose homeostasis [3].

In a systematic review and meta-analysis of 11 randomised clinical trials (RCTs) comparing surgical to non-surgical treatment of morbid obesity, bariatric surgery was associated with greater weight loss, higher remission rates of T2DM and metabolic syndrome, better lipid profiles, greater improvement in quality of life, and substantial reductions in medication requirements [5]. Furthermore, a growing number of recent RCTs in patients with T2DM, including mainly individuals with BMI >35 kg/m^2^, have consistently demonstrated superior efficacy of bariatric surgery in reducing weight and lowering glycemia, compared to a variety of medical and lifestyle interventions [6–13].

The aim of this review is to discuss the major pathophysiological mechanisms mediating weight loss and T2DM remission after bariatric surgery, summarize the clinical and biological predictors of T2DM remission after surgery, and provide an update on the short, mid- and long-term effects of bariatric surgery in obese patients with T2DM with a focus on weight loss durability, T2DM remission, improvement of cardiovascular risk factors, diabetes-related complications, mortality and survival. Recent recommendations concerning the role of bariatric surgery in the treatment algorithm of patients with T2DM are critically appraised, whereas controversial issues and knowledge gaps are further discussed.

## Main text

### Description of surgical procedures and their rationale

**Fig. 1:**
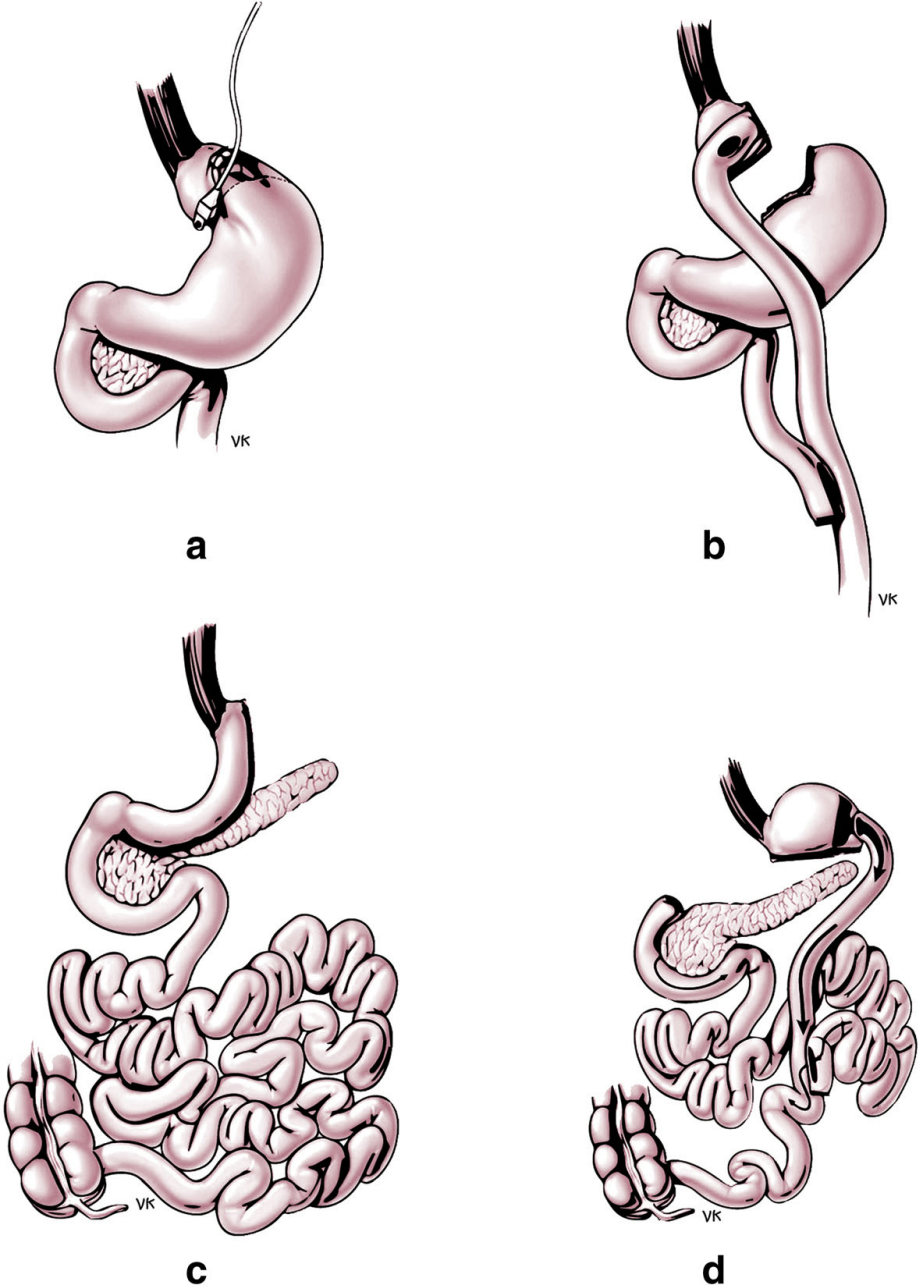
Graphical presentation of the four best established and standardized types of bariatric surgery. **a** Laparoscopic adjustable gastric banding, **b** Roux-en-y gastric bypass, **c** Vertical sleeve gastrectomy and **d** Biliopancreatic diversion

The major distinction between bariatric procedures relies on their mechanisms of action and comprises purely restrictive, malabsorptive or combination techniques. Of note, the length of the upper gastrointestinal tract bypass is thought to play a major role in determining the magnitude of weight loss and metabolic outcomes [14]. Among various techniques, the following four are the most studied in terms of safety and efficacy to promote weight loss and improve metabolic state and overall health (Fig. 1).

Laparoscopic adjustable gastric banding (LAGB) involves encircling the upper part of the stomach with a silicone adjustable band with an inflatable balloon at the inner surface connected with a port placed subcutaneously, through which pressure on the vagal afferent intraganglionic laminar endings (IGLEs) can be adjusted by adding or retracting fluid (Fig. 1a).

Roux-en-Y gastric bypass (RYGB) is a mixed technique combining both gastric and small bowel mechanisms. It includes generating a small gastric pouch (15–30 mL) on the lesser gastric curvature, which is isolated from the gastric remnant and anastomosed to the jejunum, leaving an alimentary or Roux limb of typically 100–150 cm. Bowel continuity is restored by an entero-enteric anastomosis between the excluded biliopancreatic limb and the alimentary limb (Fig. 1b). This anastomosis is usually performed 100–150 cm distal to the gastro-jejunostomy, although it has been also performed up to 250 cm distally in an attempt to create caloric malabsorption. However, most patients after RYGB become constipated, hence caloric malabsorption does not appear to play a role [14].

Sleeve gastrectomy (SG) is based on the resection of the main part of the fundus and corpus of the stomach, starting 2–8 cm proximally to the pylorus (Fig. 1c). This procedure was initially used as the first step in a two-staged approach applied in extremely obese patients, due to technical difficulties of performing bypass surgical techniques in one single session. However, significant weight loss and metabolic improvement was observed after SG alone, thus making the method very popular as a standalone procedure.

Finally, biliopancreatic diversion (BPD) with or without duodenal switch does create caloric malabsorption. This operation consists of a horizontal gastrectomy and anastomosis between the remaining stomach and the distal 250 cm of the small intestine. The bypassed duodenum, jejunum and part of the proximal ileum carry bile and pancreatic secretions and are connected to the alimentary limb 50 cm proximal to the ileocecal valve (Fig. 1d).

All operations are almost always performed laparoscopically with small operative times and low complication rates. The major limitation of LAGB is that approximately 20% of patients do not experience any changes in hunger and hence no weight loss. They often regain weight and need to undergo multiple revisional operations [15]. The major complications associated with the remaining types of surgery include physiological (dumping syndrome, chronic nutritional deficiencies, loss of bone density and kidney/gallbladder stone formation) and mechanical (abdominal pain, anastomotic stenosis) side effects [14].

### Pathophysiology of weight loss and T2DM remission after bariatric surgery

Metabolic surgery is able to induce and maintain substantial weight loss through a variety of mechanisms, including caloric restriction as a result of the anatomical remodelling of the gastrointestinal tract, increased meal-induced thermogenesis, modulation of hypothalamic neuronal circuits involved in energy balance and appetite regulation, altered taste, food preferences and eating behaviour patterns, as well as altered gut-brain signalling pathways [1, 3, 14, 16–18].

With regard to amelioration of the metabolic milieu leading to T2DM remission, the beneficial effects of metabolic surgery are mediated to a significant extent by two important factors; the hypocaloric state due to profound caloric restriction, and the significant weight loss achieved. Studies involving patients with T2DM have demonstrated that caloric restriction to the extent observed during the first 10 to 20 days after RYGB has the same immediate effect on insulin sensitivity and blood glucose levels as surgery [17]. However, there is no enhanced incretin effect with caloric restriction alone, and it is essentially impossible for people with obesity and diabetes to maintain this drastic restriction for a prolonged period of time.

There are also other well-established weight loss-independent therapeutic effects (specific to bariatric surgery), which can be observed in the early postoperative period preceding major weight loss, and cannot be reproduced by comparable diet-induced weight loss. The major pathophysiological mechanisms mediating these beneficial metabolic effects of upper gastrointestinal tract bypass surgery in particular, comprise the following: effects on multi-organ insulin sensitivity (hepatic and skeletal muscle), β-cell function, changes in bile acid (BA) composition and flow, modifications of gut microbiota, intestinal glucose metabolism, and increased metabolic activity of brown adipose tissue [16].

Whether the effects of surgery and particularly RYGB and LAGB on insulin sensitivity are totally independent of weight loss remains controversial, since a similar improvement of insulin action has been demonstrated in non-surgically-treated patients losing the same amount of weight under a comparable dietary caloric restriction [16]. On the contrary, BPD appears to exert unique effects on insulin sensitivity; studies have shown rapid clamp-assessed insulin sensitivity improvement after a minimal weight loss of <10% [16]. The short- and long-term effects of metabolic surgery on tissue-specific insulin sensitivity currently represent a highly interesting field of research. Rapid improvement of hepatic insulin sensitivity in the short-term may be a result of the sudden and profound caloric restriction, whereas the beneficial effects on skeletal muscle insulin action are observed later on (mid- and long-term effects) and are therefore predominantly driven by weight loss [1, 16].

The first phase of insulin secretion and the incretin effect, both severely impaired in patients with T2DM, are rapidly restored to normal after metabolic surgery and especially RYGB [14, 16]. The anatomical bypass of the proximal intestine and the rapid delivery of undigested food from stomach to small intestine lead to a rapid increase of circulating incretin levels [glucagon-like peptide 1 (GLP-1), glucose-dependent insulinotropic peptide (GIP)], which in turn may promote pancreatic insulin release and gradually improve β-cell function (hindgut hypothesis). Beyond incretins, additional gastrointestinal postprandial satiety hormones such as peptide YY (PYY) are significantly elevated [16, 19].

The driving force behind first-phase insulin secretion restoration is still a matter of controversy; severe caloric restriction, massive weight loss, and the robust increase in postprandial incretin secretion, may all be implicated. In favor of the first hypothesis, a study in 11 patients with T2DM who underwent caloric restriction similar to that immediately after bariatric surgery, showed beta cell function normalization, increased insulin sensitivity, restoration of first-phase insulin secretion, and, ultimately, diabetes reversal [20]. In support of the effect of weight loss, studies using intravenous glucose tolerance tests (IGTTs), in which insulin secretion is by definition incretin-independent, have also shown major improvements in early insulin secretion in both diabetic and non-diabetic bariatric patients [21, 22]. The merits of the enhanced incretin response cannot, however, be overlooked; in an elegantly conceived study in five patients who had undergone RYGB and, for medical reasons, had a gastrostomy tube placed, the administration of a glucose load through the gastrostomy (thus negating the effect of proximal gut bypass) led to a much lower incretin and insulin response than when the same load was given orally [23]. Thus, it is quite plausible that all the above putative mechanisms (and possibly others) act in concert to restore early postprandial insulin secretion in bariatric patients.

Regarding the enhanced incretin response, although the hindgut hypothesis appears quite plausible, studies have also focused on the role of the foregut (foregut hypothesis). It has been postulated that diabetogenic “anti-incretin” hormones are normally released when a meal passes through the proximal small intestine, and that rerouting food with bypass surgery reduces their secretion, thus promoting antidiabetic effects [17]. Although research points to a role of the foregut in T2DM remission after RYGB, no such hormones have yet been identified.

Bile acid (BA) metabolism has recently emerged as an additional important contributor of surgically-induced metabolic improvement with an accumulating body of evidence showing alterations in BA composition and flow after metabolic surgery. It has been postulated that weight loss after RYGB may be associated with increased fasting and postprandial serum BA levels [3, 14, 16]. However, there is no direct evidence to confirm a causal relationship between circulating BA concentrations and an improved metabolic milieu in surgical patients. Data in this field are conflicting. Contrary to RYGB, LAGB has been associated with reduced basal and postprandial serum BA concentrations, in parallel, however, with a similar improvement in β-cell function and insulin sensitivity compared to RYBG. Based on the above data, changes in BA metabolism are definitely an important player, but firm conclusions regarding the causality of this relationship and its clinical implications cannot yet be drawn.

RYGB-induced weight loss has been further associated with changes in gut microbiota, namely increased intestinal microbial diversity, as well as changes in the relative amounts of specific bacterial phyla and species [14, 16]. The major factors leading to these modifications include changes in body weight, dietary intake, nutrient flow through the intestine, gut motility, intraluminal pH and bile flow. Whether the beneficial effects of altered gut microbiota on glucose homeostasis are weight loss-independent remains unclear.

Animal data have shown that RYGB induces villus hyperplasia and increases villus height in the Roux limb, therefore leading to increased intestinal glucose uptake [16]. In human studies with positron emission tomography scans and clamps, an increased insulin-stimulated jejunal glucose uptake 6 months after RYGB in patients with and without T2DM has been shown. However, the magnitude of intestinal glucose retention after mixed meal ingestion in humans is considered to be too small to induce a clinically relevant improvement of postprandial glycemic control.

Finally, it has been proposed that the increased BA and incretin levels achieved after RYGB may upregulate the metabolic activity of residual brown adipose tissue located in small amounts in the supraclavicular fat depots in adults, and even induce browning of white adipose tissue, leading to a better metabolic state and facilitating weight loss [16].

### Clinical and biological determinants of weight loss and T2DM remission after surgery

With regard to weight loss, the preoperative postprandial gut hormone responses (incretin effect) are not prognostic for surgically-induced weight loss [14]. Interestingly, higher baseline levels of the soluble receptor for advanced glycation endproducts (sRAGE) have been associated with better weight loss after bariatric surgery (RYGB, SG, LAGB) [24].

A large number of studies have tried to explore the best biological and clinical predictors of T2DM remission after bariatric surgery. In this context, the distinction among remitters, never remitters and relapsers after initial remission is crucial. T2DM remission should be defined accurately and uniformly to facilitate comparability of results between different studies. Complete T2DM remission is defined as fasting plasma glucose <100 mg/dl and/or HbA1c <6% for at least 1 year after surgery in the absence of glucose-lowering pharmacologic treatment. Partial T2DM remission is defined as fasting plasma glucose <126 mg/dl and/or HbA1c <6.5% off antidiabetic medication for at least 1 year [15, 25]. A prolonged complete T2DM remission, extending beyond 5 years, may be viewed as operationally equivalent to cure [25]. A cautious approach is, however, warranted when using these terms, especially when proposing monitoring and treatment algorithms for T2DM. Based on data from long-term trials, the median T2DM-free interval after surgery has been estimated to be 8.3 years (RYGB), while T2DM relapse -restarting antidiabetic medication or fasting plasma glucose >126 mg/dl and/or HbA1c >6.5%- may be observed in as many as 20-30% after initial remission over a period of 6 years [26]. Data from a retrospective cohort study in a large number of patients with T2DM undergoing RYGB, show that up to 70% may experience an initial complete remission in the first 5 years, but among these, 35% may redevelop T2DM within 5 years after remission [26].

The favourable effect of surgically-induced weight loss on T2DM remission appears to be independent of initial BMI. Accumulating evidence suggests that preoperative BMI within the obese range is not able to reliably predict cardiometabolic benefits with regard to T2DM prevention and remission, incidence and mortality of cardiovascular disease [15]. A recently published meta-analysis of RCTs, controlled clinical trials and cohort studies, conducted in two distinct BMI groups (<35 and >35 kg/m^2^), revealed similar T2DM remission rates in both groups, regardless of baseline BMI [27]. Whereas preoperative BMI appears to be unhelpful as a determinant of metabolic improvement, body weight change trajectories, namely the degree of weight reduction, play a central role in predicting responders vs. non-responders [26]. In patients experiencing massive weight loss after surgery, T2DM remission rates are considerably higher compared to subjects with less profound weight loss, irrespective of baseline BMI categorization [26], suggesting that weight loss per se appears to have a dominant effect.

The International Diabetes Federation’s definition of optimal metabolic control may be more helpful, as the focus shifts from remission to long-term control of all metabolic parameters. As such, the benefits of metabolic surgery with regard to glycemia, hypertension and dyslipidemia, are equally valued. The complementary use of medication is also encouraged to enhance and maintain the metabolic benefits of surgery longer.

Beyond weight reduction, baseline T2DM duration, preoperative use of intensive insulin regimens, and poorer glycemic control, have been consistently associated with lower rates of T2DM remission and higher risk of relapse [27, 28]. Many, however, argue that these are the patients that would actually benefit most from metabolic surgery, because they can have the largest improvements in metabolic control even if they do not achieve complete hyperglycemia remission. These patients may actually have to be prioritised over those who can easily be placed into remission, as the former typically have the highest morbidity and mortality. On the contrary, a shorter T2DM duration (<8 years), lower preoperative fasting glycemia, and surgical procedures involving intestinal diversion instead of gastric only procedures may independently predict higher T2DM remission rates and lower risk of recidivism [27]. Lower baseline waist circumference may further predict a greater HbA1c reduction [27], whereas baseline visceral fat area is also associated with better metabolic outcomes, especially in Asian T2DM patients with increased visceral fat, despite only mildly elevated BMI [29]. Thus, it becomes evident that an early operation combined with better controlled baseline glycemia are expected to be beneficial, prompting a need for the early recognition of appropriate candidates for surgery [27, 28], but this should not be done at the expense of patients with long-standing and poorly controlled diabetes, where a surgical intervention will likely have dramatic and long-term beneficial effects on glycemia, even if complete remission is not achieved.

### Short- and mid-term outcomes of bariatric surgery in obese patients with T2DM

Table 1 summarizes the key findings of major RCTs studying the short- and mid-term (up to 1 and 3 years respectively) outcomes of bariatric surgery in overweight and obese patients with T2DM regarding weight loss, T2DM remission, improvement of glycemic control, and cardiovascular risk factors.

**Table 1:** Major RCTs on the short and mid-term outcomes of bariatric surgery in patients with T2DM

Reference	Study population	Intervention	Duration of follow-up	Major outcomes	Key results
Dixon et al., 2008 [30]	*N* = 60, BMI 30–40, recently diagnosed T2DM	LAGB	2 years	Weight loss, T2DM remission	↑ weight loss and T2DM remission after surgery vs. conventional diabetes treatment
Mingrone et al., 2012 [6]	*N* = 60, BMI > 35, inadequately controlled T2DM for at least 5 years	RYGB, BPD	2 years	HbA1c, T2DM remission	↑ T2DM remission after surgery vs. conventional treatment
Schauer et al., 2012 [7]	*N* = 150, poorly controlled T2DM	RYGB, SG	1 year	Weight loss, HbA1c, medication use	Weight loss RYGB > SG > IMT, ↓ medication to lower glucose, lipids and blood pressure after surgeryvs. IMT
Ikramuddin et al., 2013 [11]	*N* = 120, BMI 30–40, poorly controlled T2DM	RYGB	1 year	CVD risk factors	Better control of HbA1c, lipids and blood pressure after surgery vs. IMT
Courcoulas et al., 2014 [14]	*N* = 69, obesity grade I/II	RYGB, LAGB	1 year	Weight loss, T2DM remission	Weight loss RYGB > LAGB> LWLI, ↑ T2DM remission vs. LWLI
Halperin et al., 2014 [10]	*N* = 38, BMI 30–42, T2DM of at least 1 year	RYGB	1 year	Partial T2DM remission, CVD risk factors	↑ T2DM remission and greater improvement in blood pressure and lipids vs. IMT, equal improvement in QOL
Parikh et al., 2014 [24]	*N* = 57, BMI 30–35, T2DM	RYGB, SG, LAGB	6 months	T2DM remission, insulin resistance, medication use	↑ T2DM remission, improved HOMA-IR, ↓ HbA1c, fasting glucose and antidiabetic drugs after surgery vs. MWM
Schauer et al., 2014 [33]	*N* = 150, poorly controlled T2DM	RYGB, SG	3 years	T2DM remission, QOL, medication use	↑ T2DM remission, ↓ glucose-lowering medication, ↑QOL after surgery vs. IMT
Wentworth et al., 2014 [31]	*N* = 51, BMI 25–30, T2DM	LAGB	2 years	T2DM remission	↑ T2DM remission after LAGB vs. conventional treatment alone, acceptable adverse event profile
Courcoulas et al., 2015 [13]	*N* = 69, obesity grade I/II	RYGB, LAGB	3 years	Weight loss, T2DM remission	Weight loss RYGB > LAGB> LWLI, ↑ T2DM remission vs. LWLI
Ding et al., 2015 [32]	*N* = 45, BMI 30–45, T2DM of at least 1 year	LAGB	1 year	Glycemic endpoint comprising HbA1c and fasting glucose	Similar HbA1c reduction, weight loss at 3 months, blood pressure, lipids and CVD risk scores after LAGB and MWM (no difference)
Cummings et al., 2016 [9]	*N* = 43, BMI 30–45, T2DM	RYGB	1 year	Complete T2DM remission, medication use	↑ T2DM remission, ↓ antidiabetic drugs after surgery vs. IMT

*BMI* body mass index, *BPD* biliopancreatic diversion, *CVD* cardiovascular disease, *HbA1c* glycosylated haemoglobin, *HOMA-IR* homeostasis model assessment index for insulin resistance, *IMT* intensive medical treatment, *LAGB* laparoscopic adjustable gastric banding, *LWLI* lifestyle weight loss intervention, *MWM* medical weight management, *QOL* quality of life, *RYGB* Roux-en-Y Gastric Bypass, *SG* sleeve gastrectomy, *T2DM* type 2 diabetes mellitus

LAGB: An Australian open-label study in 60 obese patients (BMI 30–40 kg/m^2^) with recently diagnosed T2DM compared LAGB to conventional diabetes therapy with focus on weight loss and T2DM remission for a follow-up period of 2 years, and reported T2DM remission rates of 73% in the surgical group versus only 13% in the control group [30]. Weight loss after LAGB was nearly 10-fold higher (20.7% vs. 1.7%). In this study, T2DM remission was related to the extent of weight loss and lower baseline HbA1c levels. In another RCT in 50 overweight patients with T2DM randomly allocated to intensive diabetes care with or without LAGB, T2DM remission at 2 years was achieved in 50% of LAGB patients, and 8% in the control group [31]. However, in both studies, long-term efficacy and safety data are missing. In contrast with the above data, another RCT in obese patients with T2DM comparing LAGB with an intensive medical weight management program, reported similar HbA1c reductions at 3 and 12 months in both groups, similar weight loss at 3 months and similar changes in blood pressure, serum lipids, cardiovascular risk scores and patient-reported health outcomes after both interventions, thus questioning the real potential of LAGB to powerfully affect cardiometabolic outcomes in this population [32].

RYGB/BPD/SG: In an Italian, single-center, non-blinded RCT, 60 obese patients with a BMI >35 kg/m^2^ and a history of at least 5 years of poorly controlled T2DM, were randomized to either conventional medical treatment or RYGB/BPD [6]. T2DM remission at 2 years was specified as fasting plasma glucose <100 mg/dl and HbA1c <6.5% without pharmacologic therapy for at least 1 year, and was achieved by none of the medically-treated patients, 75% of RYGB-treated patients and 95% of BPD-treated patients. In multivariate regression analysis, age, gender, baseline BMI, T2DM duration and body weight changes were not significant predictors of T2DM remission at 2 years or improvement of glycemic control at 1 and 3 months [6]. Authors concluded that bariatric surgery can definitely result in better short-term metabolic control in poorly controlled T2DM patients than high-standard medical therapy, but baseline adiposity and weight loss could not predict improved metabolic outcomes.

In a similarly designed single-center RCT (STAMPEDE) comparing intensive medical treatment (IMT) with medical therapy plus RYGB or SG in 150 severely obese patients with poorly controlled T2DM (baseline HbA1c 9%), the percentage of patients with complete T2DM remission (HbA1c <6%) at 1-year follow-up was 42% in the RYGB and 37% in the SG group, compared to only 12% in the IMT group [7]. Extended follow-up to 3 years post-surgery (mid-term outcomes), showed T2DM remission rates of only 5% in the IMT group, compared to 38% in the RYGB and 24% in the SG group, and a significantly lower use of glucose-lowering medications in the surgical groups [33]. An important difference in this study was that patients were allowed to stay on oral hypoglycemic agents to allow them to achieve an HbA1c < 6%, suggesting that the benefit of surgery can be further enhanced with the use of appropriate medication.

RYGB was further compared to IMT in another RCT (Diabetes Surgery Study) in terms of cardiovascular risk factors including T2DM, hypertension and hyperlipidemia [11]. This study enrolled 120 obese participants with T2DM of at least 6 months’ duration and poor glycemic control. A composite triple endpoint of HbA1c <7% + LDL-cholesterol <100 mg/dl + systolic blood pressure < 130 mmHg was achieved in significantly more patients in the RYGB group compared to IMT both at 1 and 2 years of follow-up (43-49% vs 14-19%). Of note, these outcomes were mainly attributable to weight loss and predominantly driven by improved glycemic control, as shown by regression analyses [11]. With regard to T2DM remission, the comparison between RYGB and IMT reveals higher remission rates at 1 year, approaching 90% in some studies, alongside a greater impact on quality of life and amelioration of insulin resistance, subclinical inflammation and cardiovascular comorbidities [34]. Data from the CROSSROADS RCT comparing RYGB vs. IMT in terms of T2DM remission and diabetes medication requirements are in line with the above [9].

RYGB appears to be significantly more effective than LAGB in terms of weight loss and T2DM remission in short- and mid-term studies. In a three-arm single-center RCT including 69 patients with grade I and II obesity and concomitant T2DM, RYGB and LAGB were compared to an intensive lifestyle weight loss intervention (LWLI) at 1 and 3 years of follow-up [12]. The gradient for weight loss and complete and partial T2DM remission rates at 1 and 3 years of follow-up was RYGB >LAGB >LWLI [12, 13].

### Long-term outcomes of bariatric surgery in obese patients with T2DM

Table 2 summarizes the key findings of major studies investigating long-term (beyond 5 years of follow-up) outcomes of bariatric surgery in obese patients with T2DM regarding sustainability and maintenance of weight loss, T2DM prevention, remission and relapse, cardiometabolic risk factors, micro- and macrovascular complications, and mortality.

**Table 2:** Major studies on the long-term outcomes of bariatric surgery in obese patients with T2DM

Reference	Study population	Intervention	Duration of follow-up	Major outcomes	Key results
Sjöström et al., 2004 [35]	SOS cohort	LAGB, VBG, RYGB	10 years	CVD risk factors (remission/prevention)	↑ recovery from T2DM, dyslipidemia, HTN and hyperuricemia, ↓ incidence of T2DM and lipid disorders after surgery
Adams et al., 2007 [49]	*N* = 9949	RYGB	7.1 years	Total and cause-specific mortality	↓ T2DM-, cancer- and CHD-related mortality, ↑ mortality related to accidents and suicide after RYGB
Sjöström et al., 2007 [48]	SOS cohort	LAGB, VBG, RYGB	10.9 years	Overall mortality	↓ CVD, cancer and overall mortality after surgery vs. usual conventional care
Iaconelli et al., 2011 [39]	*N* = 110, BMI > 35, newly diagnosed decompensated T2DM	BPD	10 years	Micro- and macrovascular complications, renal function, T2DM remission	↑ recovery from microalbuminuria, ↓ CHD probability after surgery vs. medical arm
Adams et al., 2012 [36]	*N* = 1156, BMI > 35	RYGB	6 years	Weight loss, T2DM remission	Superior weight loss maintenance, ↑ T2DM remission, ↓ T2DM incidence after surgery vs. control group
Sjöström et al., 2012 [45]	SOS cohort	LAGB, VBG, RYGB	14.7 years	Stroke, myocardial infarction	↓ CVD incidence and mortality after surgery vs. usual conventional care
Arterburn et al., 2013 [26]	*N* = 4434, inadequately controlled T2DM	RYGB	10 years	T2DM remission	68% T2DM remission within 5 years post-RYGB, 35% relapse within 5 years after initial remission, median duration of remission 8.3 years
Brethauer et al., 2013 [44]	*N* = 217	LAGB, RYGB, SG	6 years	T2DM remission, cardiometabolic comorbidities	25% T2DM remission after surgery, 19% T2DM relapse after initial remission, up to 80% control of cardiometabolic risk factors
Sjöström et al., 2014 [40]	SOS cohort	LAGB, VBG, RYGB	18 years	T2DM remission, micro- and macrovascular complications	↓ incidence of micro- and macrovascular T2DM-related complications
Arterburn et al., 2015 [47]	*N* = 2500, mainly ♂	LAGB, RYGB, SG	14 years	Mortality/ survival	↓ all-cause mortality after surgery
Mingrone et al., 2015 [8]	*N* = 60, BMI > 35, inadequately controlled T2DM for at least 5 years	RYGB, BPD	5 years	T2DM remission, CVD risk, medication use, QOL, diabetes-related complications	↑T2DM remission but existing risk of relapse,↓lipids, CVD risk, medication use and major complications after surgery vs. conventional diabetes treatment
Schauer et al., 2017 [38]	*N* = 150, BMI 27–43, poorly controlled T2DM	RYGB, SG	5 years	HbA1c <6% with or without medication	Metabolic endpoint met by 29% of RYGB, 23% of SG and 5% of IMT group, superior weight loss, better lipid profile, ↓ use of insulin, ↑ QOL after surgery

*BMI* body mass index, *BPD* biliopancreatic diversion, *CHD* coronary heart disease, *CVD* cardiovascular disease, *HbA1c* glycosylated haemoglobin, *HTN* hypertension, *IMT* intensive medical treatment, *LAGB* laparoscopic adjustable gastric banding, *QOL* quality of life, *RYGB* Roux-en-Y Gastric Bypass, *SG* sleeve gastrectomy, *SOS* Swedish Obese Subjects, *T2DM* type 2 diabetes mellitus, *VBG* vertical banded gastroplasty

The SOS (Swedish Obese Subjects) study represents a landmark in the field, investigating the short-, mid- and long-term effects of surgery up to 20 years postoperatively. It is a prospective matched cohort study conducted in Sweden with a recruitment period of 1987–2001 [34].

#### Durability of weight loss

In the SOS study, body weight remained reduced by 16% 10 years after surgery, while it increased by 1.6% in the matched control group [35]. In a prospective Utah-based study in over 1000 obese patients undergoing RYGB, weight loss reached 28% up to 6 years after surgery, and RYGB achieved superior weight loss maintenance compared to control interventions; 94% of RYGB-treated patients maintained at least 20% of their initial weight loss at 2 years, and 76% maintained at least 20% at 6 years [36]. These findings are consistent with a “set point change” for weight in the subcortical areas of the brain, which suggests that postoperatively, the subcortical areas of the brain are “under the impression” that patients are 25–30% overweight and thus the bodyweight is brought down to the new level where the set point is now established.

#### T2DM prevention, remission, relapse

A prespecified secondary endpoint of the SOS study was the incidence of new-onset T2DM during 15 years of follow-up in a large number of obese patients undergoing bariatric surgery vs matched obese controls. New-onset T2DM was reported in 6.8 cases per 1000 patient-years in the surgical group compared to 28.4 cases per 1000 patient-years in the control group [37]. Of interest, the effect of bariatric surgery to prevent T2DM by reducing incidence, was influenced by the presence of impaired fasting glucose at baseline (prediabetes), but not by preoperative BMI. This finding provided ground for addressing the need to revisit the current BMI-centric eligibility criteria for bariatric surgery [37].

In the 5-year follow-up study by Mingrone et al. comparing RYGB/BPD to conventional medical treatment in poorly controlled obese patients with long-standing T2DM, 50% of surgically-treated patients maintained partial T2DM remission at 5 years, whereas 0% achieved complete T2DM remission at 5 years [8]. Recurrence of T2DM was observed in half of the patients who achieved a 2-year T2DM remission after RYGB and one third of patients who achieved a 2-year remission after BPD, and was unrelated to the magnitude of weight loss. This was one of the first studies clearly indicating that continued monitoring of glycemic control after bariatric surgery is warranted, despite initial T2DM remission, due to the existing risk of hyperglycemia recurrence.

In the recently published 5-year follow-up of the STAMPEDE trial, the primary endpoint of achieving HbA1c <6% with or without antidiabetic medication was achieved by 29% of RYGB and 23% of SG patients, compared to only 5% of the IMT group [38]. This effect was seen in parallel with superior weight loss, better lipid profile, lower use of insulin, and improved quality of life after both surgical procedures [38].

#### Micro- and macrovascular complications of T2DM

In the study by Mingrone et al., major diabetes-related complications, including acute myocardial infarction, were reported in nearly one third of medically-treated patients compared to only one case in the RYGB group and 0 cases in the BPD group [8].

Dramatically reduced renal and cardiovascular complications have been documented up to 10 years after BPD compared to non-surgical approaches, indicating long-term benefits of this type of surgery on T2DM-related life-threatening complications. In support of this, a case-controlled Italian study in obese patients with newly-diagnosed T2DM showed that mean glomerular filtration rate (GFR) declined by nearly 50% in the medical arm, and increased by 13.6% in the surgical arm during a follow-up of 10 years after BPD. After 10 years, all BPD patients had their microalbuminuria restored to normal, whereas microalbuminuria progressed to macroalbuminuria in conventionally treated patients [39].

In a long-term observational follow-up of the SOS study (extending up to 15 years), the cumulative incidence of microvascular diabetes-related complications was two-fold higher in the control group versus the surgical group (41.8 vs 20.6 cases per 1000 person-years), whereas the respective incidence of macrovascular diabetes-related complications (stroke and/or myocardial infarction) was 44.2 cases in the control, and 31.7 cases in the surgical group [40]. Prospective interventional studies suggest that diabetic nephropathy may improve within 1 year, but retinopathy and neuropathy remain stable for 1 year and may require longer to show improvements [41–43].

#### Cardiovascular risk factors

The SOS study showed that the rates of T2DM, dyslipidemia, hypertension and hyperuricemia resolution were significantly higher and the respective incidence rates were significantly lower 10 years after surgery, compared to the control group [35]. More specifically, the rate of recovery for hypertriglyceridemia was 80%, low HDL cholesterol 73%, high LDL 72%, and hypertension 62% over a period of at least 6 years post-surgery [44]. The incidence of cardiovascular events was reduced by 33%, but only in patients with hyperinsulinemia, while BMI was not able to predict any benefit [45]. Low-grade systemic inflammation, which characterizes obesity and diabetes, is also improved after all bariatric operations [46].

#### Survival and mortality

Bariatric surgery has been associated with improved long-term survival and reduced overall and cause-specific mortality related to T2DM, cardiovascular disease and cancer [47, 48]. Reduced all-cause mortality at 5 years and up to 10 years (mid- and long-term) has been reported in both male and female populations [49], and patients with T2DM or hyperinsulinemia apparently benefit more. Bariatric surgery has been also associated with a reduced incidence of cancer, although only in females [50].

### Comparative evaluation of different surgical procedures on cardiometabolic outcomes

The gradient of efficacy for the four well-established procedures for weight loss and T2DM remission is: BPD > RYGB > SG > LAGB. The opposite gradient has been proposed for their comparative safety [15].

Among the four operations, RYGB appears to have the most favourable risk-benefit profile in most patients with T2DM. Although longer-term data are needed, current information suggests that SG is quite effective, resulting in excellent weight loss and major improvements in T2DM, at least in the short to medium term (1–3 years). It is a valuable option for morbidly obese patients with T2DM, especially those for whom concerns exist about the risk of operations involving bowel diversion [51]. LAGB is effective in improving glycemia in patients with obesity and T2DM, to the degree that it causes weight loss. It is associated, however, with a greater risk of reoperation due to failure or band-related complications. Although clinical evidence suggests that BPD may be the most effective procedure in terms of glycemic control and weight loss, it is associated with a significant risk of nutritional deficiencies, making its risk-benefit profile less favourable. BPD should be primarily reserved for patients with extreme obesity (BMI >60 kg/m^2^) in centres with significant expertise to monitor these patients in the long term [15].

### Potential for less invasive, device-based, techniques

The EndoBarrier® gastrointestinal liner, or else the duodenal/jejunal bypass sleeve technique (DJBS), is less invasive compared to bariatric surgery and totally reversible. It can be applied as a bridge to induce rapid weight loss prior to surgery and has shown satisfactory short-term weight loss results in multicentric randomized efficacy studies [52]. It is placed endoscopically (mean procedure time: 35 min for implantation, approximately 17 min for explantation). The majority of patients experience at least one adverse event during the first week post-implantation, mainly mild abdominal pain and nausea. A systematic review and meta-analysis of five RCTs and ten observational studies evaluating its efficacy and safety in obese patients with and without T2DM suggested that it may provide better weight loss than diet modification alone [53]. However, differences in glycemic endpoints such as HbA1c and fasting plasma glucose failed to reach statistical significance. Major adverse events included abdominal pain, nausea, and vomiting, while no fatal outcomes were reported [53].

Saline-filled intragastric balloon devices are reversible endoscopic approaches, designed to occupy stomach volume and reduce food intake. A dual balloon system device (DBS) plus diet and exercise was compared to a lifestyle intervention alone in the REDUCE trial, a prospective RCT of DBS for the treatment of obesity: 326 patients with grade I and II obesity were randomized to endoscopic DBS plus diet or sham endoscopy plus diet. The devices were extracted 6 months after implantation. DBS was found to be significantly more effective than diet in promoting weight loss with a relatively acceptable safety profile (mainly gastric ulcers) [54].

### Critical appraisal of current metabolic surgery guidelines for diabetes treatment

Recently, a number of leading international diabetes organizations issued new guidelines for the treatment of obese patients with T2DM, and integrated metabolic surgery into the proposed treatment algorithm after extensive evidence appraisal and broad consensus. These guidelines were developed in the second Diabetes Surgery Summit (DSS-II), and were endorsed by numerous medical and scientific societies worldwide [15]. According to these, bariatric surgery should be recommended to treat T2DM in obese patients with a BMI ≥40 kg/m^2^ (grade III obesity), regardless of glycemic control or complexity of glucose-lowering regimens, and also in patients with a BMI between 35 and 39.9 kg/m^2^ (grade II obesity), if hyperglycemia cannot be adequately controlled despite optimal lifestyle and medical treatment. Furthermore, metabolic surgery should be considered as an option to treat T2DM in obese patients with a BMI between 30 and 34.9 kg/m^2^ (grade I obesity), if adequate glycemic control is not achieved despite maximally intensified treatment with oral or injectable medications [15].

In this joint statement, a critical approach towards the benefits and limitations of metabolic surgery was adopted and a number of controversial issues were raised, highlighting the complexities in the field. Some of these issues are summarized below:

- Long-term (beyond 10 years) efficacy and safety data are lacking.
- Factors predicting T2DM remission and relapse after surgery are still incompletely characterized.
- There is still insufficient evidence to clearly define cut-off values for T2DM duration or laboratory markers that might be able to quantitatively predict T2DM remission over time.
- Available studies include a modest number of patients with a BMI between 30 and 35 kg/m^2^.
- There are limited comparative (head-to-head) data for distinct surgical interventions in terms of cardiometabolic outcomes.
- Data for T2DM-related complications, cancer and mortality, representing hard and clinically relevant endpoints, can be extrapolated only from non-randomized studies. RCTs for these outcomes are warranted.
- There are no studies investigating the role of multimodal therapy with integration of pharmaceutical and surgical treatment strategies to optimize outcomes for T2DM patients, in terms of inducing and maintaining T2DM remission as well as lowering the risk of complications and comorbidities.
- It is necessary to identify more reliable clinical and biological markers, which can be applied in an accurate definition of T2DM remission and cure.
- Cost-effectiveness data are pending.
- Optimal intervention time for a durable T2DM remission still remains elusive.
- The relationship between the duration of T2DM remission and the incidence of micro- and macrovascular complications remains unclear.

## Conclusions

The gastrointestinal tract represents a meaningful target to treat T2DM. Surgical operations with intestinal diversion have consistently shown the capacity to improve glucose homeostasis by modulating gut hormones, beyond reducing energy intake and body weight. LAGB, SG, RYGB and BPD are the four best standardized surgical procedures.

The beneficial metabolic effects of surgery are partly mediated by weight loss and severe energy restriction, but there are also weight loss-independent mechanisms. Foremost of these are effects on multi-organ insulin sensitivity, β-cell function and incretin response, changes in BA composition and flow, modifications of gut microbiota, intestinal glucose metabolism, and increased metabolic activity of brown adipose tissue.

Many studies have tried to explore the best biological and clinical predictors of T2DM remission after surgery. The distinction between responders, never remitters and relapsers after initial remission is crucial. A shorter T2DM duration, better preoperative glycemic control, lower baseline HbA1c and waist circumference, and profound postsurgical weight loss, have been all associated with higher rates of T2DM remission and lower risk of relapse, but the greatest benefit may actually occur in patients with the largest reduction in HbA1c, even if they stay on medication and do not meet remission criteria. Interestingly, preoperative BMI is of no predictive utility for metabolic outcomes.

Many RCTs and high-quality prospective matched cohort studies have consistently shown beneficial effects of bariatric surgery in obese patients with T2DM. In the short and medium term (up to 3 years post-surgery), a significant amount of weight is lost, T2DM may completely regress, and cardiometabolic risk factors are dramatically improved. In the long term (beyond 5 years), surgery may achieve durable weight loss, prevent T2DM and cancer, improve overall glycemic control (although T2DM might recur in a considerable percentage of patients), and reduce total and cause-specific mortality. The gradient of efficacy for the four established procedures for weight loss and T2DM remission is: BPD > RYGB > SG > LAGB.

According to recently released guidelines and recommendations, metabolic surgery should be recommended in appropriate candidates with class III obesity, regardless of glycemic control or glucose-lowering regimens, and in patients with class II obesity with inadequately controlled T2DM despite optimal therapy, but additionally in patients with class I obesity and inadequately controlled hyperglycemia despite optimal medical treatment.

In spite of the well-established role of metabolic surgery as an additional therapeutic tool against T2DM, a number of issues still remain to be resolved; the lack of long-term efficacy and safety data, the incomplete characterization of T2DM remission determinants, the paucity of studies investigating multimodal therapies (combined surgical and pharmacological interventions), and the lack of RCTs with diabetes-related hard endpoints, such as diabetic nephropathy and retinopathy, cardiovascular disease and mortality. For metabolic surgery to become part of daily diabetes care, the surgical and non-surgical communities will need to objectively look at the data and acknowledge the strengths, weaknesses and opportunities, because, although there is a wealth of new and high-quality science in the field, the number of patients receiving surgery as a treatment for diabetes remains disappointingly low.

## Abbreviations

BA: Bile acid
BMI: Body mass index
BPD: Biliopancreatic diversion
DBS: Dual Balloon System device
DJBS: Duodenal/Jejunal Bypass Sleeve technique
DSS: Diabetes surgery summit
GIP: Glucose-dependent insulinotropic peptide
GLP-1: Glucagon-Like Peptide 1
IGLEs: Intraganglionic laminar endings
IMT: Intensive medical treatment
IVGTT: Intravenous glucose tolerance test
LAGB: Laparoscopic adjustable gastric banding
LWLI: Lifestyle weight loss intervention
PYY: Peptide YY
RCT: Randomised clinical trial
RYGB: Roux-en-Y gastric Bypass
SG: Sleeve gastrectomy
sRAGE: soluble Receptor for Advanced Glycation Endproducts
T2DM: Type 2 diabetes mellitus
